# F67. NEUROCOGNITION IN 7-YEAR-OLD CHILDREN OF PARENTS WITH SCHIZOPHRENIA OR BIPOLAR DISORDER

**DOI:** 10.1093/schbul/sby017.598

**Published:** 2018-04-01

**Authors:** Nicoline Hemager, Kerstin Plessen, Anne Amalie Thorup, Camilla J Christiani, Ditte Ellersgaard, Katrine Søborg Spang, Birgitte Klee Burton, Aja Greve, Ditte L Gantriis, Anne Søndergaard, Maja Gregersen, Ole Mors, Merete Nordentoft, Jens Richardt Møllegaard Jepsen

**Affiliations:** 1 Mental Health Centre Copenhagen, Child and Adolescent Mental Health Centre, University Hospital Lausanne; 3 Child and Adolescent Mental Health Center, Capital Region of Denmark; 4 Research Unit at Mental Health Center, The Lundbeck Foundation Initiative for Integrative Psychiatric Research (iPSYCH); 5 Aarhus University Hospital; 6 Aarhus University Hospital, The Lundbeck Foundation Initiative for Integrative Psychiatric Research (iPSYCH)

## Abstract

**Background:**

Children of parents with schizophrenia or bipolar disorder display neurocognitive deficits. However, studies of schizophrenia offspring and bipolar offspring at the same age are lacking. The objective was to compare neurocognitive abilities in 7-year-old children of parents with schizophrenia or bipolar disorder with neurocognitive abilities in children of parents without these disorders.

**Methods:**

In this nationwide cohort study we assessed 522 7-year-old children (schizophrenia offspring: N=202, bipolar offspring: N=120, and controls=200) with a detailed and well validated neurocognitive test battery. We compared the neurocognitive test scores of the three study groups.

**Results:**

Children of parents with schizophrenia showed neurocognitive deficits, whereas children of parents with bipolar disorder displayed neurocognitive abilities comparable to the control group.

**Discussion:**

Neurocognitive deficits are numerous in 7-year-old children of parents with schizophrenia, which supports the neurodevelopmental model of schizophrenia. Unimpaired neurocognitive abilities in children of parents with bipolar disorder indicate different neurodevelopmental manifestations in these high risk populations at this early age. Our results call for early identification of schizophrenia offspring with cognitive dysfunctions.

